# Exome sequencing revealed comparable frequencies of *RNF43* and *BRAF* mutations in Middle Eastern colorectal cancer

**DOI:** 10.1038/s41598-022-17449-9

**Published:** 2022-07-30

**Authors:** Abdul Khalid Siraj, Rong Bu, Tariq Masoodi, Sandeep Kumar Parvathareddy, Kaleem Iqbal, Wael Al-Haqawi, Hassan Al-Dossari, Saud Azam, Zeeshan Qadri, Padmanaban Annaiyappanaidu, Fouad Al-Dayel, Khawla Sami Al-Kuraya

**Affiliations:** 1 Human Cancer Genomic Research, Research Center, King Faisal Specialist Hospital and Research Center, MBC#98-16, P.O. Box 3354, Riyadh, 11211 Saudi Arabia; 2 Department of Pathology, King Faisal Specialist Hospital and Research Center, Riyadh, Saudi Arabia

**Keywords:** Cancer genetics, Colorectal cancer

## Abstract

Mutation-induced activation of *Wnt*-β Catenin signaling pathway is frequent in CRC. The E3 ubiquitin ligase, *RNF43*, has been reported to negatively regulate the *Wnt* signaling pathway and *RNF43* mutations are frequently seen in CRC. However, its role in Middle Eastern CRC remains unclear. Therefore, we employed Exome and Sanger sequencing technology to assess the frequency of *RNF43* mutations and its association with other clinico-pathological features in Middle Eastern CRC. *RNF43* mutations were found in 5.9% (13/220) of CRC cases and was inversely correlated to *APC* and *TP53* mutations. A strong association of *RNF43* mutations with right sided and sporadic microsatellite instable (MSI) CRC was observed. No association was identified between *RNF43* mutation and other clinico-pathological features including *BRAF* mutation, age, tumor histological subtype, tumor grade or patients’ prognosis. Multivariate logistic regression analysis revealed that MSI status and wild type *APC* were independent predictor of *RNF43* mutation. We conclude that *RNF43* mutations occur in Middle Eastern CRC at comparable frequencies with *BRAF* mutations and represent a distinct molecular subtype which further enhances our understanding of how different mutational subsets of *Wnt* tumor suppressor genes link to distinct tumor characteristics, which might be considered for treatment strategies for CRC patients.

## Introduction

Colorectal cancer (CRC) is the second leading cause of cancer-related deaths worldwide^1,2^. Increased awareness and surveillance have led to decreased incidence of CRC in Western countries, in contrast to the Middle East, where the incidence of CRC is on the rise^3–6^. In Saudi Arabia, CRC ranks first among the most frequent malignancies in men^7^, representing a huge health burden in this part of the world. Consequently, identifying new prognostic markers and therapeutic targets for CRC in this population are highly needed to improve our understanding of cancer occurrence and disease progression.

Mutation induced activation of *Wnt* signaling pathway is a well-known driver event in CRC^8,9^. Emerging evidence suggests that specific mutations in *Wnt* pathway could have different functional and phenotypic ramifications^9,10^. E3 ubiquitin-protein ligase, *RNF43*, negatively regulates the *Wnt* pathway^11^. Inactivation of *RNF43* through *RNF43* mutations can lead to permanent activation of *Wnt* pathway in cancer cells^12^. Recent studies have shown that *RNF43* mutation is a key mutational target in sporadic microsatellite unstable CRC^13–15^.

Interestingly, *RNF43* mutations are shown to be associated with distinct tumor locations and subtypes. *RNF43* mutations are enriched in microsatellite unstable tumors and right sided CRC^16,17^. Recent work showed that *RNF43* is associated with aggressive tumor biology along with *BRAF* mutation in right sided CRC^18^, further supporting a distinct pathogenic mechanism and regional preference for *Wnt* pathway alterations. Moreover, it has been reported that *RNF43* mutation was seen more frequently in sporadic microsatellite unstable (MSI) CRC than in hereditary MSI, which could suggest that sporadic MSI face more selective pressure for *RNF43* inactivation^14,19^.

However, all these studies have been conducted on CRC from different ethnic backgrounds. Data about the role of *RNF43* in Middle Eastern CRC is not known. Therefore, we conducted this study to investigate the role of somatic *RNF43* mutation in a large cohort of 220 cases using exome sequencing and Sanger sequencing to identify the prevalence, clinico-pathological association and molecular correlation in Middle Eastern CRC.

## Materials and methods

### Patient selection and tumor samples

Archival samples from 220 CRC patients diagnosed between 2000 and 2015 at King Faisal Specialist Hospital and Research Center (Riyadh, Saudi Arabia) were included in the study. Clinico-pathological data were collected from patient medical records, which are summarized in Table 1.

**Table 1:** Clinico-pathological variables for the patient cohort (n = 220).

Clinico-pathological parameter	n (%)
**Age**	
Median	46.3
Range	13.0–90.0
**Gender**	
Male	102 (46.4)
Female	118 (53.6)
**Histological subtype**	
Adenocarcinoma	190 (86.4)
Mucinous carcinoma	30 (13.6)
**Histological grade**	
Well differentiated	24 (10.9)
Moderately differentiated	152 (69.1)
Poorly differentiated	39 (17.7)
Unknown	5 (2.3)
**Tumor site**	
Left	167 (75.9)
Right	53 (24.1)
**pT**	
T1	7 (3.2)
T2	22 (10.0)
T3	152 (69.1)
T4	29 (13.2)
Unknown	10 (4.5)
**pN**	
N0	104 (47.3)
N1	63 (28.6)
N2	42 (19.1)
Nx	11 (5.0)
**pM**	
M0	181 (82.3)
M1	39 (17.7)
**TNM stage**	
I	26 (11.8)
II	75 (34.1)
III	79 (35.9)
IV	39 (17.7)
Unknown	1 (0.5)
**MMR status**	
dMMR	73 (33.2)
pMMR	147 (66.8)

### DNA isolation

DNA samples were extracted from formalin-fixed and paraffin-embedded (FFPE) CRC tumor tissues utilizing Gentra DNA Isolation Kit (Gentra, Minneapolis, MN, USA) according to the manufacturer’s protocols as elaborated in the previous studies^20^.

### Whole-exome and targeted capture sequencing

Whole exome sequencing (WES) was performed on 113 CRC cases using SureSelectXT Target Enrichment by Illumina Novaseq 6000. Quality metrics were performed on raw data using FastQC and aligned to human reference genome (hg19) using Burrows-Wheeler Aligner (BWA)^21^. The generated bam files were marked for PCR duplicates; local realignment was carried out and to obtain high quality base calls, base-quality recalibration were performed using Picard (http://broadinstitute.github.io/picard/) and GATK^22^ tools respectively.

Somatic mutation calling was performed by MuTect2^23^ and mutations were annotated with different databases using ANNOVAR^24^. The single nucleotide variants (SNVs) and indels that passed the standard MuTect2 filters were processed for further analysis, and the somatic variants with minor allele frequency (MAF) of > 0.01 in dbSNP, the NHLBI exome sequencing project, 1000 Genomes and our in-house exome database of ~ 800 normals were removed from the analysis. All the mutations were also checked and viewed using Integrated Genomics Viewer (IGV) to filter out false positives.

### Sanger sequencing analysis

**Figure 1:**
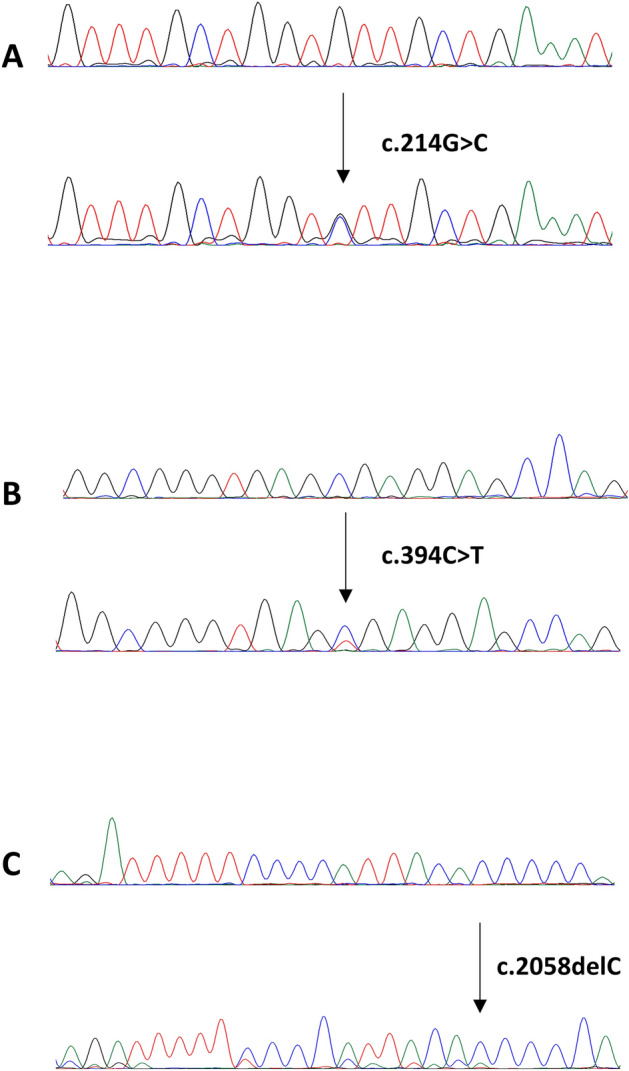
Electropherogram of three *RNF43* representative mutations identified CRC cases. Upper traces represent normal sequences while lower traces show mutated sequences. (**A**) Missense mutation, (**B**) stopgain mutation, (**C**) frameshift mutation.

Sanger sequencing technology was utilized to sequence entire coding and splicing regions of exons 2, 4, 8 and 9 in *RNF43* on 107 CRC cases. The pathogenic mutations detected by Exome sequencing/targeted capture sequencing analysis were further confirmed by Sanger sequencing analysis. Primer 3 online software was utilized to design the primers (available upon request). PCR and Sanger sequencing analysis were carried out as described previously^25^. Reference sequences were downloaded from the NCBI GenBank and sequencing results were compared with the reference sequences by Mutation Surveyor V4.04 (Soft Genetics, LLC, State College, PA) (Fig. 1).

We assessed the clinical and molecular features from patients harboring *RNF43* mutations, using OMS guidelines^26^ as well as Bethesda and Amsterdam clinical guidelines^27^ for CRC, to characterize them as serrated polyposis syndrome or other syndromes.

### Tissue microarray construction & immunohistochemistry

Tissue microarray (TMA) format was utilized for immunohistochemical analysis of samples. Construction of TMA was done as described previously^28^. Briefly, representative tumor regions from each donor tissue block were chosen and tissue cylinders with a diameter of 0.6 mm were punched and brought into recipient paraffin block with the help of a modified semiautomatic robotic precision instrument (Beecher Instruments, Wood-land, WI, USA). Two cores of CRC were arrayed from each case.

Tissue microarray slides were processed and stained manually. The immunohistochemistry (IHC) protocol was followed as mentioned before^29^. For antigen retrieval, Dako (Dako Denmark A/S, Glostrup, Denmark) Target Retrieval Solution pH 9.0 (Catalog number S2367) was used, and the slides were placed in Pascal pressure cooker for 8 min at 120 °C. The primary antibodies were diluted in a 1% solution of bovine serum albumin in phosphate buffered saline (PBS) and incubated overnight at room temperature. Primary antibodies used and their dilutions are listed in Supplementary Table S1. The Dako Envision Plus System kit was used as the secondary detection system with Diaminobenzidine (DAB) as chromogen. All slides were counterstained with hematoxylin, dehydrated, cleared and mounted. Negative controls included replacement of the primary antibody with no reacting antibodies of the same species. Only fresh cut slides were stained simultaneously to minimize the influence of slide aging and maximize reproducibility of the experiment.

**Figure 2:**
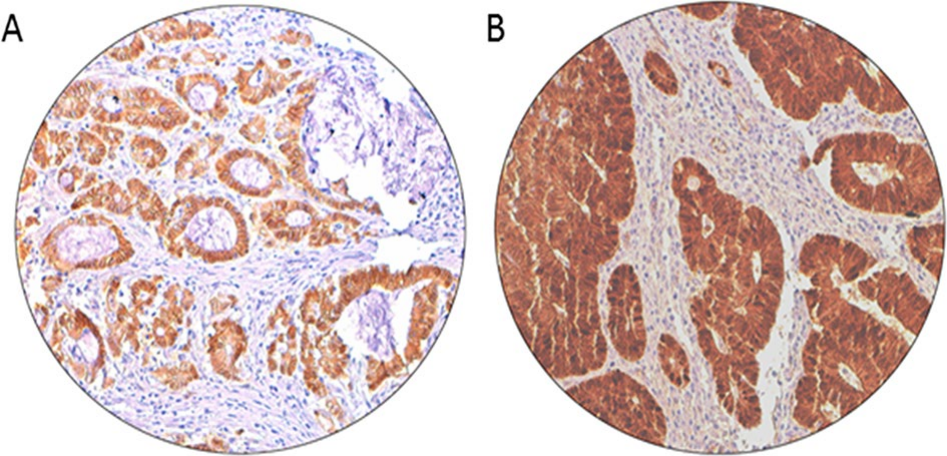
β-catenin immunohistochemical staining in colorectal carcinoma. Representative examples of tumors showing (**A**) absent nuclear expression and (**B**) high nuclear expression (right panel) of β-catenin. (×20/0.70 objective on an Olympus BX 51 microscope. (Olympus America Inc, Center Valley, PA, USA).

Evaluation of mismatch repair protein staining was performed as described previously^30^. Briefly, MMR protein expression was evaluated using *MSH2*, *MSH6*, *MLH1* and *PMS2* proteins. Details of the primary antibodies used are provided in Supplementary Table S1. Tumor was classified as deficient MMR (dMMR) if any of the four proteins showed loss of staining in cancer with concurrent positive staining in the nuclei of normal epithelial cells. Otherwise, they were classified as proficient MMR (pMMR). β-catenin scoring was performed as described previously^31^. Briefly, β-catenin expression was considered to be positive when nuclear staining intensity was moderate or strong (Fig. 2).

IHC scoring was done by two pathologists, blinded to the clinico-pathological characteristics. Discordant scores were reviewed together to achieve agreement.

### Statistical analysis

Contingency table analysis and Chi square tests were used to study the relationship between clinico-pathological variables and *RNF43* mutation. Multivariate analysis was performed using logistic regression model, after adjusting for clinico-pathological variables like age, gender, histology, stage, grade, site of tumor, MMR status, *APC* mutation and *TP53* mutation. The limit of significance for all analyses was defined as p value of < 0.05; two-sided tests were used in these calculations. The JMP14.0 (SAS Institute, Inc., Cary, NC) software package was used for data analyses.

### Ethics approval

All procedures performed in studies involving human participants were in accordance with the ethical standards of the institutional research committee and with the 1964 Helsinki declaration and its later amendments or comparable ethical standards under the project (RAC # 2190016 dated 08 October 2019) by Research Advisory Council (RAC) of King Faisal Specialist Hospital and Research Center.

### Consent to participate

Institutional Review Board of King Faisal Specialist Hospital and Research Centre provided ethical approval for the current study. Research Advisory Council (RAC) granted waiver of informed consent for use of retrospective patient case data and archival tissue samples under project RAC# 2190016.

### Consent for publication

All authors have read and approved the submitted manuscript. The manuscript has not been submitted nor published elsewhere.

## Results

### Patient characteristics

Median age of the study cohort was 46.3 years (range = 13–90 years) with a male:female ratio of 1:1.2. Majority of the cases were adenocarcinoma (86.4%), whereas mucinous CRC accounted for 13.6% of the cases. most of the tumors were moderately differentiated (69.1%) and left sided (75.9%). Lymph node metastasis was noted in 47.7% (105/220) of CRCs and distant metastasis was seen in 17.7% (39/220). 33.2% (73/220) were dMMR by IHC (Table 1).

### Mutations in known driver genes

In our entire cohort of 220 cases, the mutations in known driver genes including *APC*, *TP53*, *KRAS*, *BRAF* and *NRAS* were observed in 58% (128/220), 51% (112/220), 46% (102/220), 4% (8/220) and 3% (6/220) respectively (Supplementary Fig. 1). The frequency of cases showing more than one *APC* mutations was 43.7% (56/128) including 45 cases of double mutation, nine cases of triple mutations and two cases of quadruple mutations. Most of the mutations in *APC* gene were truncating including stopgain (52%), frameshift (40%) and only 8% missense mutations. The mutation classification in *TP53* gene include missense (68%), frameshift (10%), stop gain (18%), inframe (2%) and splicing (2%). No significant association was seen between *RNF43* and *BRAF* mutations since only one case was identified to carry mutation in *RNF43* and *BRAF* genes (7.7%).

### *RNF43* mutations identified in CRC and their clinico-pathological associations

In our study, 15 *RNF43* mutations were identified in 13 CRC cases (5.9%), ten cases detected by WES and three cases detected by Sanger sequencing, including frameshift mutation in six cases, stop gain mutation in four cases, missense mutation in four cases and splicing mutation in one case. Two cases harbored two *RNF43* mutations respectively (Table 2).

**Table 2:** *RNF43* mutations in discovery and secondary cohorts of CRC.

Ca. no.	Mutation	Coverage	Type of mutation	Exons
1	c.1248G > GA:p.416W > W/X	Exome	Stop gain	Ex 9
2	c.252 + 1G > A	Exome	Splicing	Ex 2
3	c.156delC:p.P52fs	Exome	Frameshift deletion	Ex 2
4	c.224_225delAT:p.I75fs	Exome	Frameshift deletion	Ex 2
	c.394C > CT:p.132R > R/X^a^	Exome	Stop gain	Ex 4
5	c.856C > CT:p.286R > R/W	Exome	Missense	Ex 8
6	c.394C > CT:p.132R > R/X	Exome	Stop gain	Ex 4
7	c.1855C > CA:p.619L > L/I	Exome	Missense	Ex 9
8	c.158 T > TA:p.53L > L/X	Exome	Stop gain	Ex 2
9	c.2058delC:p.P686fs	Exome	Frameshift deletion	Ex 9
10	c.1976dupG:p.P660Sfs	Exome	Frameshift insertion	Ex 9
11	c.148G > GC:p.50V > V/L	Sanger	Missense	Ex 2
	c.214G > GC:p.72V > V/L^a^	Sanger	Missense	Ex 2
12	c.1543dupGG:p.516D > G	Sanger	Frameshift insertion	Ex 9
13	c.131_139delinsTTTAAAAAGCTG	Sanger	Frameshift deletion	Ex 2

^a^Same case carried two mutations.

**Figure 3:**
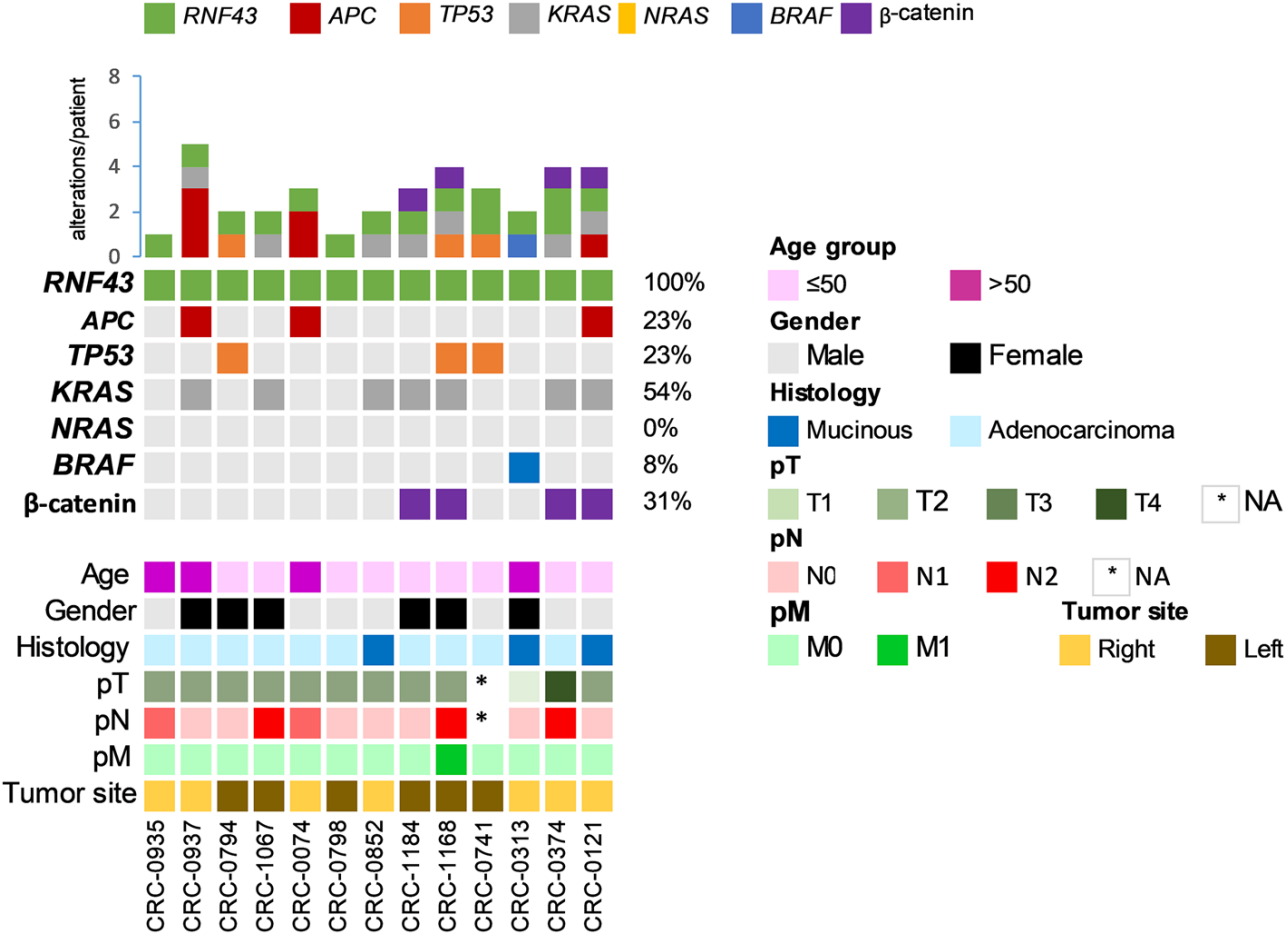
Mutations in known driver genes along with β-catenin expression in *RNF43* positive cases (n = 13). The top panel displays the number of mutations identified across tumors, whereas the right panel show the proportion of mutations/gene across each group. Clinical details are included for each patient.

*RNF43* mutation was found to be significantly associated with right-sided tumors (p = 0.0170) and dMMR (p = 0.0008). 53.9% (7/13) of the *RNF43* mutant cases were right sided tumors and 76.9% (10/13) were dMMR. We also found an association between *RNF43* mutation and *APC* wildtype (p = 0.0079) as well as *TP53* wildtype (p = 0.0341) CRC, with 76.9% (10/13) of *RNF43* mutant CRCs being *APC* wildtype and *TP53* wildtype (Table 3). However, no association was between *RNF43* mutation and nuclear β-catenin expression (Fig. 3). Interestingly, on multivariate logistic regression analysis, we found that dMMR (Odds ratio = 5.43; 95% confidence interval = 1.12–26.32; p = 0.0356) and *APC* wildtype (Odds ratio = 4.77; 95% confidence interval = 1.51–19.77; p = 0.0312) were independent predictors of *RNF43* mutation (Table 4).

**Table 3:** Correlation of *RNF43* mutation with clinico-pathological parameters in colorectal carcinoma.

	Total		*RNF43* mutant		*RNF43* wildtype		p value
	n	%	n	%	n	%	
**Total number of cases**	220		13	5.9	207	94.1	
**Age**							
≤ 50 years	158	71.8	9	69.2	149	72.0	0.8321
> 50 years	62	28.2	4	30.8	58	28.0	
**Sex**							
Male	102	46.4	7	53.8	95	45.9	0.5777
Female	118	53.6	6	46.2	112	54.1	
**Tumour site**							
Left colon	167	75.9	6	46.2	161	77.8	0.0170*
Right colon	53	24.1	7	53.8	46	22.2	
**Histological type**							
Adenocarcinoma	190	86.4	10	76.9	180	87.0	0.3417
Mucinous carcinoma	30	13.6	3	23.1	27	13.0	
**pT**							
T1	7	3.3	0	0.0	7	3.5	0.7044
T2	22	10.5	1	8.3	21	10.6	
T3	152	72.4	10	83.3	142	71.7	
T4	29	13.8	1	8.3	28	14.1	
**pN**							
N0	104	49.8	7	58.3	97	49.2	0.5410
N1	63	30.1	2	16.7	61	31.0	
N2	42	20.1	3	25.0	39	19.8	
**pM**							
M0	181	82.3	12	92.3	169	81.6	0.2839
M1	39	17.7	1	8.3	38	19.2	
**Tumour stage**							
I	26	11.9	1	7.7	25	12.1	0.6217
II	75	34.2	6	46.1	69	33.5	
III	79	36.1	5	38.5	74	35.9	
IV	39	17.8	1	7.7	38	18.5	
**Differentiation**							
Well differentiated	24	11.2	3	25.0	21	10.3	0.3775
Moderate differentiated	152	70.7	7	58.3	145	71.4	
Poor differentiated	39	18.1	2	16.7	37	18.2	
**MMR-IHC**							
dMMR	73	33.2	10	76.9	63	30.4	0.0008*
pMMR	147	66.8	3	23.1	144	69.6	
***BRAF* mutation**							
Present	8	3.6	1	7.7	7	3.4	0.4770
Absent	212	96.4	12	92.3	200	96.6	
***APC* mutation**							
Present	128	58.2	3	23.1	125	60.4	0.0079*
Absent	92	41.8	10	76.9	82	39.6	
***TP53* mutation**							
Present	112	50.9	3	23.1	109	52.7	0.0341*
Absent	108	49.1	10	76.9	98	47.3	
***KRAS* mutation**							
Present	102	46.4	7	53.8	95	45.9	0.5777
Absent	118	53.6	6	46.2	112	54.1	
***NRAS* mutation**							
Present	6	2.7	0	0.0	6	2.9	0.3892
Absent	214	97.3	13	100.0	201	97.1	
**Nuclear β catenin IHC**							
High	96	46.4	4	30.8	92	47.4	0.2365
Low	111	53.6	9	69.2	102	52.6	

*Significant p value.

**Table 4:** Multivariate logistic regression analysis to assess relationship between *RNF43* mutation and clinico-pathological characteristics.

Clinico-pathological variables	Odds ratio	95% Confidence interval	p-value
**Age**			
> 50 years (vs. ≤ 50 years)	0.59	0.12–3.04	0.5299
**Gender**			
Male (vs. female)	1.39	0.34–5.64	0.6454
**Histology**			
Mucinous (vs. adenocarcinoma)	0.36	0.03–4.26	0.4173
**Tumor site**			
Right (vs. left)	1.70	0.32–9.05	0.5364
**Tumor grade**			
Grade 3 (vs. Grade 1 & 2)	0.83	0.12–5.88	0.8483
**Stage**			
IV (vs I–III)	0.44	0.04–4.27	0.4762
**MMR status**			
dMMR (vs. pMMR)	5.95	1.08–32.76	0.0405*
***APC* mutation**			
Wild type (vs. mutant)	6.60	1.23–35.51	0.0280*
***TP53* mutation**			
Wild type (vs. mutant)	0.94	0.18–4.91	0.9399

*Significant p value.

## Discussion

A screening for mutations in 220 primary CRCs identified *RNF43* coding for ring domain E3 ubiquitin-protein ligase is mutated in Middle Eastern CRC. Our analysis revealed that 5.9% of the Middle Eastern CRC patients had *RNF43* mutations. This is similar to what has been reported in the largest existing *RNF43* study of Seeber et al. (6.1%)^32^. Similar frequency of *RNF43* mutant cases has also been observed in TCGA cohort where *RNF43* mutations were seen in about 5.7% (12/212) of CRC patients^33^. Different studies have reported higher incidence of *RNF43* mutations in CRC^14,18^. This differences could be attributed to the sample size differences, technical artifacts and ethnic backgrounds of CRC patient analyzed.

Consistent with previous reports^14,15,34,35^, multivariate analysis revealed that dMMR tumors and wild type *APC* were independently associated with *RNF43* in this population. Importantly, *RNF43* ranked fourth in mutational frequency to *APC* (58.2%; 128/220), *TP53* (50.9%; 112/220), *KRAS* (46.4%; 102/220) and at comparable frequency with *BRAF* (3.6%; 8/220) suggesting an important role of this gene in Middle Eastern CRC.

Similar to previous studies, *RNF43* mutations were found strongly enriched with MSI-linked hypermutated CRC tumors^14,36,37^. Despite the shared dMMR phenotype between *RNF43* mutations and Lynch syndrome, we could not identify any *RNF43* mutations in the Lynch syndrome dMMR cancers. *RNF43* mutations were seen mainly in sporadic MSI cancers. This difference in *RNF43* mutations between hereditary and sporadic dMMR CRC (which also have been documented previously)^19^ could be due to the presence of other mutations in *Wnt* pathway genes in hereditary MSI CRC tumors.

Furthermore, we observed inverse association between *RNF43* mutations and *APC* as well as *TP53* mutations. This suggests the presence of relationship between type of *Wnt* pathway mutations and the biological context of CRC tumors.

*RNF43* mutations were not associated with tumor grade, stage or specific histology. In addition, no significant correlation was found between *RNF43* and *BRAF* mutations, contrary to previous reports^18,36^. This discrepancy could be attributed to the known low rate of *BRAF* mutations in CRC from this ethnicity and rare prevalence of serrated adenoma as precursor of Middle Eastern CRC^38^. Previous reports have linked *RNF43* mutations with serrated pathway of CRC development that originates from serrated polyp enriched for activating *BRAF* mutations and mismatch repair (MMR) gene mutations^15,36,39^. In addition, *RNF43* mutations were shown previously to be a late event that drive the progression of sessile serrated adenoma^36,40^. Therefore, it was hypothesized that *RNF43* mutations exerts lower impact on *Wnt* pathway activation. To test this hypothesis, (as a way to examine *Wnt* pathway activation) we examined nuclear β-catenin accumulation via IHC in the entire cohort, and tested the association with *RNF43* mutations. We found no correlation between *RNF43* mutations and nuclear β-catenin level which is supportive of the notion that *RNF43* does not display prominent role in *Wnt* pathway activation. These results must be interpreted carefully in light of some limitations of this study with regards to sample size and lack of copy number variation data. Further larger studies including entire chromosomal instability data are needed to confirm the association between *RNF43* and nuclear β-catenin accumulation. The co-occurrence of *RNF43* and *BRAF* mutations was not seen in our study contrary to previous report which showed significant association between *RNF43* mutations and *BRAF* mutations in CRC due to their involvement in MSI and CIMP pathways in colorectal carcinogenesis^18^. This result could be attributed to the low *BRAF* mutations identified in CRC patients from Middle Eastern population as previously reported^38^.

Interestingly, *RNF43* mutations were found to be significantly enriched in right sided CRCs, which has also previously been reported in other studies^16,17,32,41^, and suggests that *RNF43* mutations are associated with distinct primary tumor locations within the colon, further supporting regional differences for *Wnt* pathway alterations.

Despite the interesting findings of this study, we acknowledge that this analysis has some limitations. First, this retrospective analysis was performed at a single institution. Second, the number of patients with *RNF43* mutations are limited. Third, this study was conducted on patients from specific ethnicity. Therefore, future study from larger cohort, multi-institution and different ethnicity is needed.

In conclusion, we have found that dysregulated *Wnt* signaling and mutations in the tumor suppressor *RNF43* are involved in Middle Eastern CRC and is representative of a CRC subset with distinct characteristics.

## Supplementary Information

Supplementary Figure S1.

Supplementary Table S1.

## Data Availability

The datasets generated during and/or analyzed during the current study are available from the corresponding author on reasonable request.
