# Supplementary figures and images for: Exome sequencing revealed comparable frequencies of RNF43 and BRAF mutations in Middle Eastern colorectal cancer
Source: Sci Rep. 2022 Jul 30;12:13098. doi: 10.1038/s41598-022-17449-9 (PMC9338933; doi:10.1038/s41598-022-17449-9)

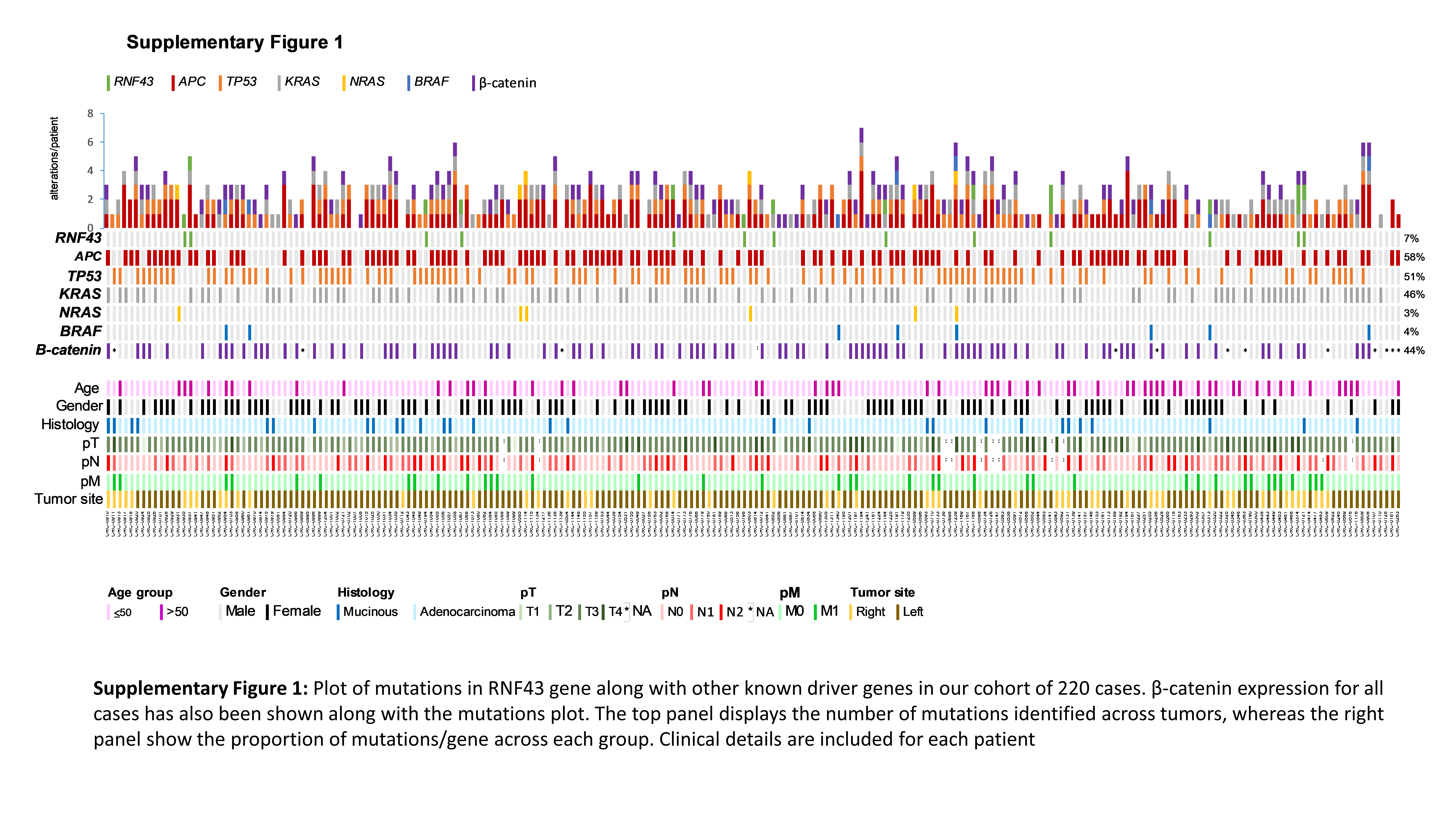

Supplement: Supplementary file 1 — Supplementary Figure S1. [file 41598_2022_17449_MOESM1_ESM.tif]
