# Supplementary material for: Exome sequencing revealed comparable frequencies of RNF43 and BRAF mutations in Middle Eastern colorectal cancer
Source: Sci Rep. 2022 Jul 30;12:13098. doi: 10.1038/s41598-022-17449-9 (PMC9338933; doi:10.1038/s41598-022-17449-9)
Supplement: Supplementary file 2 — Supplementary Table S1. [file 41598_2022_17449_MOESM2_ESM.docx]

**Supplementary Table S1.** Antibodies used for TMA IHC analysis

| **Antibody** | **Clone** | **Company** | **Dilution*** | **Retrieval** | **Detection Kit** |
| --- | --- | --- | --- | --- | --- |
| MLH1 | G168-15 | BD Pharmingen | 1:50 | pH 9 | Dako EnVision+ |
| MSH2 | FE11 | CalBiochem | 1:100 | pH 9 | Dako EnVision+ |
| MSH6 | 44 | BD Transduction Laboratories | 1:100 | pH 9 | Dako EnVision+ |
| PMS2 | C-20 | Santa Cruz Biotechnology | 1:100 | pH 9 | Dako EnVision+ |
| β - catenin | 14 | BD Transduction Laboratories | 1:5000 | pH 6 | Dako EnVision+ |

*Overnight incubation
